# Modifiable Risk Factors and Hearing Loss: The National Health and Nutrition Examination Survey 1999–2020

**DOI:** 10.1111/coa.70153

**Published:** 2026-07-31

**Authors:** Yulu Zheng, Tingting Li, Zhiyuan Wu, Yahong Zhu, Zheng Guo, Xingang Li

**Affiliations:** ^1^ Centre for Precision Health Edith Cowan University Joondalup WA Australia; ^2^ Medical Research Center Jinan Maternity and Child Care Hospital Affiliated to Shandong First Medical University Jinan Shandong China; ^3^ School of Science Edith Cowan University Joondalup WA Australia; ^4^ Nutrition and Innovation Research Institute Edith Cowan University Joondalup WA Australia

**Keywords:** hearing loss, modifiable risk factors, population attributable fraction, prevalence ratio

## Abstract

**Objective:**

To explore the associations between major modifiable risk factors and hearing loss.

**Methods:**

This serial cross‐sectional study was conducted within the National Health and Nutrition Examination Survey 1999–2020 in the United States. Associations were assessed using modified Poisson regression and within‐sample population attributable fraction (PAF).

**Results:**

Eight thousand seven hundred and seventy adults aged 40–85 years were included in the analyses, the mean (SD) age was 58.0 (11.7) years, with 4002 men (45.6%). Hearing loss was associated with low educational attainment with prevalence ratio 1.23, 95% confidence interval (1.15, 1.31); low income 1.11 (1.04, 1.19); diabetes 1.10 (1.03, 1.18); HDL‐C dyslipidaemia 1.13 (1.06, 1.21); obesity 1.08 (1.01, 1.15) and tobacco use 1.11 (1.04, 1.19). Within our analysis, over 25% of hearing loss cases were attributed to modifiable risk factors, with low education or income, HDL‐C dyslipidaemia, obesity and tobacco use being the leading contributors, though relative rankings varied slightly in survey‐weighted analyses.

**Conclusion:**

Hearing promotion policies should prioritise related modifiable factors to reduce the burden of hearing loss.

## Key Points

- This study explores modifiable risk factors linked to hearing loss in adults aged 40–85 years.
- Over 25% of hearing loss cases are attributed to modifiable risk factors.
- Low education or income, HDL‐C dyslipidaemia, obesity and tobacco use contribute most to hearing loss.
- Age, sex and ethnicity variations in risk factor associations for hearing loss were examined.
- Modified Poisson regression and population‐attributable fractions ensured robust estimates.

## 1 Introduction

Age‐related hearing loss is a common and permanent auditory impairment among middle‐aged and older adults [1]. With an ageing population, its prevalence and related health issues are expected to rise significantly in the coming decades [2]. Currently, about 1.5 billion people worldwide experience hearing loss, which is expected to reach 2.5 billion by 2050 [3]. Unaddressed hearing loss can negatively impact quality of life, increasing the odds of falling [4], depression [5], cognitive decline [6], dementia [7] and even higher mortality rates in the elderly [8]. Identifying modifiable risk factors is crucial for preventing and mitigating auditory impairment.

The primary causes of age‐related hearing loss include vascular degeneration, auditory nerve damage, cochlear dysfunction, mitochondrial DNA mutation and metabolic diseases [1, 2, 9]. Vascular dysfunction, particularly, in the spiral ligaments or cochlea, is common in older individuals with hearing loss. Systemic chronic inflammation is also thought to play a key role in age‐related hearing loss [10]. Microangiopathy and systemic chronic inflammation are often linked to metabolic or age‐related diseases (e.g., hypertension, dyslipidaemia, obesity, diabetes and cognitive decline), which may accelerate hearing loss progression [11]. Previous studies have mainly focused on one or two metabolic risk factors for hearing loss, like hypertension, hyperlipidaemia, obesity and hyperglycaemia. In addition, other attributable factors related to socioeconomic, psychological, behavioural and environmental risks may also contribute to hearing loss [5, 12].

Hence, this study aims to quantify and compare the associations and population‐attributable fractions of 10 common modifiable risk factors for age‐related hearing loss among middle‐aged and older adults. This investigation also explores whether these associations differ by age, sex and ethnicity.

## 2 Materials and Methods

### 2.1 Study Design and Participants

This study was a serial cross‐sectional analysis using data from the National Health and Nutrition Examination Survey (NHANES). NHANES is an ongoing annual survey since 1999, designed to collect data from a representative sample of the US civilian, noninstitutionalised population. Detailed information on NHANES design and recruitment is available elsewhere [13]. The data, released in 2‐year intervals, include demographic, anthropometric, laboratory‐based and questionnaire‐based information. For this study, we analysed data from the 1999–2020 NHANES waves (excluding 2013–2014 when audiometric data were unavailable).

We excluded individuals without audiometry tests or assessment on modifiable risk factors. We further excluded 1644 participants with hearing thresholds > 70 dB hearing level (dB HL) at any tested frequency (0.5–8 kHz) as very high thresholds may reflect equipment limitations or measurement error and profound hearing loss may have distinct etiologies (congenital, ototoxic, traumatic) not representative of age‐related hearing loss in the general population. Finally, our analytic cohort included 8770 adults aged 40 to 85 years, with available data on audiometric tests and modifiable risk factors (Figure 1).

**FIGURE 1:**
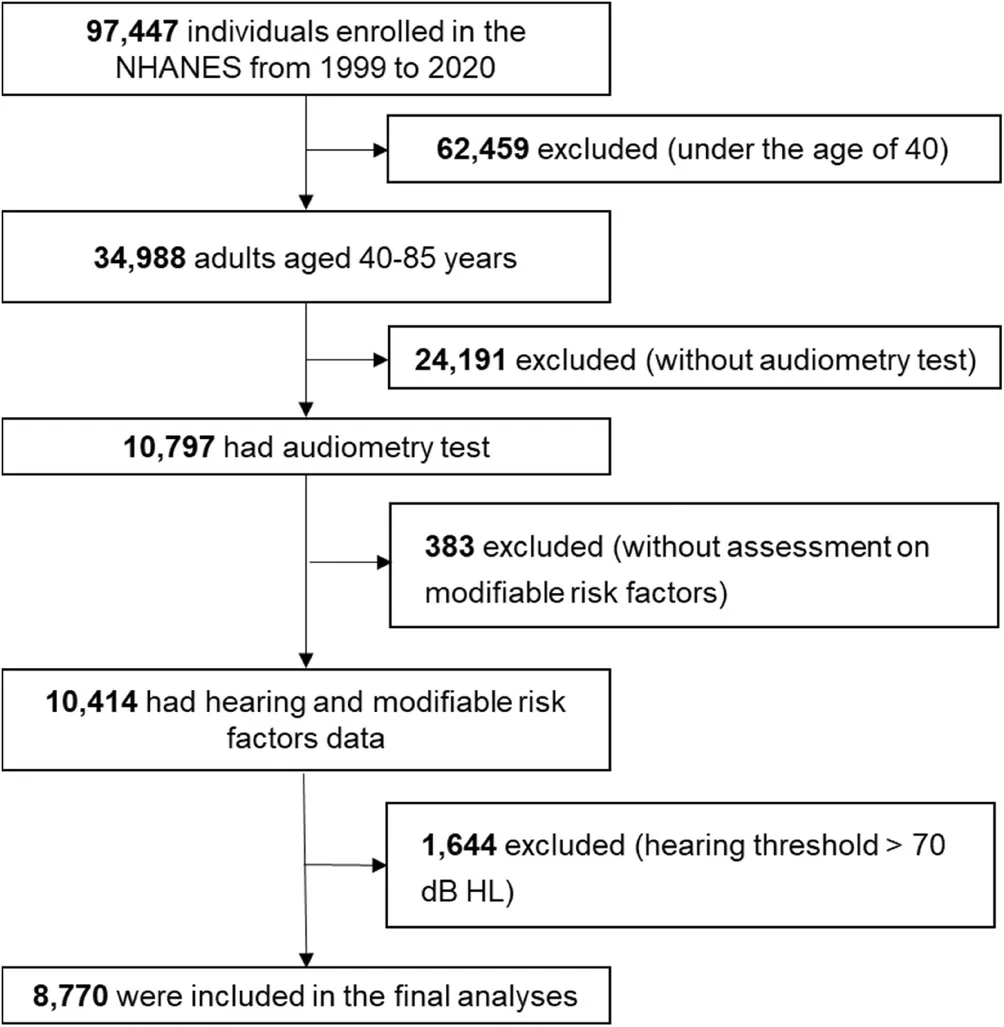
Participant flowchart. dB HL, decibel hearing level; NHANES, National Health and Nutrition Examination Survey.

### 2.2 Hearing Assessment

Audiometry was performed in sound‐attenuating booths by trained examiners following NHANES protocols. Hearing thresholds were tested in both ears at frequencies 0.5, 1, 2, 3, 4, 6 and 8 kHz, using a modified Hughson Westlake procedure. A four‐frequency (0.5, 1, 2, 4 kHz) pure‐tone average (PTA) was calculated for the better‐hearing ear, with higher scores indicating worse hearing. High‐frequency (3, 4, 6, 8 kHz) and low‐frequency (0.5, 1, 2 kHz) PTAs were also calculated for the better‐hearing ear. Hearing loss was defined as the four‐frequency PTA ≥ 20 dB HL (better ear) [14].

### 2.3 Modifiable Health Risk Factors

We considered 10 modifiable risk factors as exposures as they are amenable to intervention at both individual and population levels through public health policy, behavioural change or medical management. Those included socio‐economic and psychological risks (i.e., low education, low income and depression); metabolic risks (i.e., hypertension, diabetes, dyslipidaemia and abdominal obesity); behavioural risks (i.e., tobacco use and alcohol drinking) and environmental risk (i.e., noise exposure). Definitions, measurements and categorisations of these factors, based on widely recognised and applied standards, are detailed in Table 1.

**TABLE 1:** Definitions, measurements and categorisations of 10 modifiable risk factors.

Modifiable risk factor	Definition or measurement	Risk category	Reference category
Socio‐economic and psychological risks			
Education	Self‐reported education is classified as low (less than 12th grade), intermediate (high school or GED education) or high (college or university education).	Low education	Intermediate education or high education
Household annual income	Self‐reported household annual income was classified as low (< $35 000), middle ($35 000–$99 999) or high (≥ $100 000).	Low household income	High or middle household income
Depression	Symptoms of depression were reported based on the Depression Screener Questionnaire, where nine symptom questions are scored from ‘0’ (not at all) to ‘3’ (nearly every day). Symptoms consistent with depression were defined as a score > 10.	Symptoms consistent with depression	No depression
Metabolic risks			
Hypertension	Hypertension was defined as a systolic blood pressure > 140/90 mmHg, a self‐reported history of hypertension or treatment with antihypertensive medications.	Definition of hypertension was met	Definition of hypertension was not met
Diabetes	Diabetes was defined as fasting glucose ≥ 7 mmol/L or self‐reported history of diabetes or treatment for diabetes.	Definition of diabetes was met	Definition of diabetes was not met
HDL‐C dyslipidaemia	HDL‐C dyslipidaemia is defined as HDL cholesterol < 1.0 mmol/L for men and < 1.3 mmol/L for women.	Definition of HDL‐C dyslipidaemia was met	Definition of HDL‐C dyslipidaemia was not met
Obesity	BMI = weight (kg)/height (m)^2^ WHtR = circumference of the waist/circumference of the height	BMI ≥ 30 and WHtR ≥ 0.5	BMI < 30 and WHtR < 0.5
Behavioural risks			
Tobacco use	Self‐reported tobacco use evaluated via NHANES participant questionnaire; categorised as every day, some days or never.	Every day or some days	No tobacco use
Alcohol drinking	Had at least 12 alcohol drinks per year or in participant's entire life?	Alcohol drinks ≥ 12 per year or in participant's entire life	Alcohol drinks < 12 per year or in participant's entire life
Environmental risk			
Noise exposure	Self‐reported ongoing occupational noise exposure.	Ongoing occupational noise exposure	No occupational noise exposure

Abbreviations: BMI, body mass index; GED, general educational development; HDL‐C, high‐density lipoprotein cholesterol; NHANES, National Health and Nutrition Examination Survey; WHtR, waist to height ratio.

### 2.4 Statistical Analysis

A sample size of over 8770 provided sufficient power (with 1 − *β* over 85%) to detect differences in hearing loss across risk factor groups. Participant characteristics were described for the overall population. As the rare disease assumption is violated in this cross‐sectional design [15], modified Poisson regression models with sandwich variances were employed to ensure robust estimations for the associations of major modifiable risk factors with hearing loss. Prevalence ratios (PRs) with 95% confidence intervals (CIs) were calculated using a single model adjusted for all risk factors, age, sex and ethnicity. Risk factors were dichotomised into ‘non‐exposed reference’ and ‘exposed risk’ categories, with detailed PR calculations in the Supporting Information.

Population‐level risks of hearing loss attributable to modifiable risk factors were assessed using average PAF and 95% CIs via the ‘averisk’ package in R software version 4.1.0 (R Foundation for Statistical Computing, Vienna, Austria). PAFs for the 10 modifiable risk factors were derived from a single model adjusted for age, sex, ethnicity and mutual adjustment for all modifiable risk factors. Given the various possible orders by which the modifiable risk factors could be eliminated from the population, the average PAF is defined as the expected proportion of hearing loss preventable by the additional elimination of the risk factor of interest when the risk factors are sequentially eliminated from the population in a random order [16]. Total PAFs included only modifiable risk factors with positive PAF estimates, with details provided in the Supporting Information.

Missing data for income, depression, blood pressure, cholesterol, fasting glucose, tobacco use and alcohol consumption were imputed using the Multivariate Imputation via Chained Equations (*MICE*) package in R software version 4.1.0. Assuming data were missing at random, classification and regression trees were applied for imputation over 5 imputations with 20 iterations, ensuring convergence for all variables. The diagnostic statistics per estimate were computed by Rubin's rules [17].

To verify the robustness of the results, multiple sensitivity analyses were conducted for the association analyses between modifiable risk factors and hearing loss. First, we repeated the analyses for hearing loss at high‐ and low‐frequency PTA. Second, we re‐analysed the association between modifiable risk factors and moderate hearing loss, defined as the four‐frequency PTA > 40 dB HL. Third, we stratified the analyses by age. Fourth, we repeated the analyses by stratifying sex. Fifth, we stratified the analyses by ethnicity (i.e., Mexican American, other Hispanic, non‐Hispanic White, non‐Hispanic Black and other Race). Sixth, we calculated the adjusted PAF, with the adjustments for all modifiable risk factors, age, sex and ethnicity. Moreover, we repeated our primary association analysis by including all participants (*n* = 10 414) regardless of hearing threshold severity. Last, weighted PAF estimates were additionally calculated using NHANES complex survey weights, incorporating strata and primary sampling units. Weighted PRs were estimated using survey‐weighted modified Poisson regression models.

The analyses were performed using R software version 4.1.0. *p* < 0.05 (two‐tailed) was considered statistically significant throughout. All analyses were unweighted as the primary aim was to estimate associations (PRs). This approach is consistent with analytical studies prioritising internal validity.

## 3 Results

### 3.1 Participant Characteristics

Among 8770 participants aged 40–85 years, the mean (standard deviation, SD) age was 58.0 (11.7) years; 4768 were women (54.4%) and 4002 were men (45.6%). Of the participants, 2416 (27.5%) had hearing loss at four‐frequency PTA, 4946 (56.4%) at high‐frequency PTA and 1663 (19.0%) at low‐frequency PTA.

Participants with hearing loss were older, had lower education and income and showed higher prevalence of hypertension, diabetes, high‐density lipoprotein cholesterol (HDL‐C) dyslipidaemia and obesity compared to those with normal hearing. Detailed characteristics are provided in Table 2.

**TABLE 2:** Characteristics of the study population by hearing status.

Variables^a^	Hearing status	
	Normal	Hearing loss^b^
Number of participants, %	6354 (72.5)	2416 (27.5)
Demographic characteristics		
Age	54.74 (10.28)	66.49 (10.72)^c^
Sex (male, %)	2733 (43.0)	1269 (52.5)^c^
Ethnicity, %		
Mexican American	1014 (16.0)	346 (14.3)^c^
Other Hispanic	571 (9.0)	215 (8.9)
Non‐Hispanic White	2451 (38.6)	1232 (51.0)
Non‐Hispanic Black	1629 (25.6)	441 (18.3)
Other race	689 (10.8)	182 (7.5)
Socio‐economic risk factors		
Education, %		
Less than 12th grade	1493 (23.5)	829 (34.3)^c^
High school or GED education	1368 (21.5)	620 (25.7)
College or University education	3493 (55.0)	967 (40.0)
Annual family income, %		
< $35 000	2725 (42.9)	1316 (54.5)^c^
$35 000–$99 999	2877 (45.3)	942 (39.0)
≥ $100 000	752 (11.8)	158 (6.5)
Depression, %	528 (8.3)	224 (9.3)
Metabolic risk factors		
Hypertension, %	3199 (50.3)	1611 (66.7)^c^
SBP (mmHg)	127.58 (18.71)	133.46 (20.48)^c^
DBP (mmHg)	73.44 (12.30)	69.64 (14.23)^c^
Type 2 diabetes	1074 (16.9)	680 (28.1)^c^
FBG (mmol/L), median [IQR]	5.66 [5.26, 6.38]	5.83 [5.32, 6.56]^c^
HDL‐C dyslipidaemia, %	1921 (30.2)	749 (31.0)
HDL‐C, median [IQR]	1.34 [1.11, 1.63]	1.29 [1.06, 1.60]^c^
Obesity, %	2546 (40.1)	975 (40.4)
BMI	29.56 (6.80)	29.52 (6.42)
WHtR	0.60 (0.09)	0.62 (0.09)^c^
Behavioural risk factors		
Tobacco use, %	2326 (36.6)	778 (32.2)^c^
Alcohol drinking, %	5220 (82.2)	1746 (72.3)^c^
Environmental risk factors		
Noise exposure, %	432 (6.8)	118 (4.9)^d^

Abbreviations: BMI, body mass index; DBP, diastolic blood pressure; FBG, fasting blood glucose; GED, general educational development; HDL‐C, high‐density lipoprotein cholesterol; SBP, systolic blood pressure; WHtR, waist to height ratio.

^a^ Comparisons of groups are based on *t*‐test for normally distributed continuous variables; Mann–Whitney *U* test for non‐normally distributed continuous variables; *χ* ^2^ test for qualitative variables.

^b^ Defined as normal (PTA < 20 dB HL) and as slight to profound hearing loss (PTA ≥ 20 dB HL) for the better‐hearing ear at four frequency PTA (mean hearing threshold across 0.5, 1, 2 and 4 kHz).

^c^ *p* < 0.001.

^d^ *p* < 0.05.

### 3.2 Associations of Modifiable Health Risk Factors With Hearing Loss

Compared to the high educational attainment group (non‐exposed reference category), the low educational attainment group (exposed risk category) had a 23% (PR 1.23, 95% CI, 1.15–1.31) higher prevalence of hearing loss. Additionally, the prevalence of hearing loss was higher in multiple ‘exposed risk group’ compared with ‘non‐exposed reference group’, such as low income (PR 1.11, 95% CI, 1.04–1.19); diabetes (PR 1.10, 95% CI, 1.03–1.18); HDL‐C dyslipidaemia (PR 1.13, 95% CI, 1.06–1.21); obesity (PR 1.08, 95% CI, 1.01–1.15); and tobacco use (PR 1.11, 95% CI, 1.04–1.19). Detailed associations of hearing loss with modifiable risk factors are presented in Figure 2.

**FIGURE 2:**
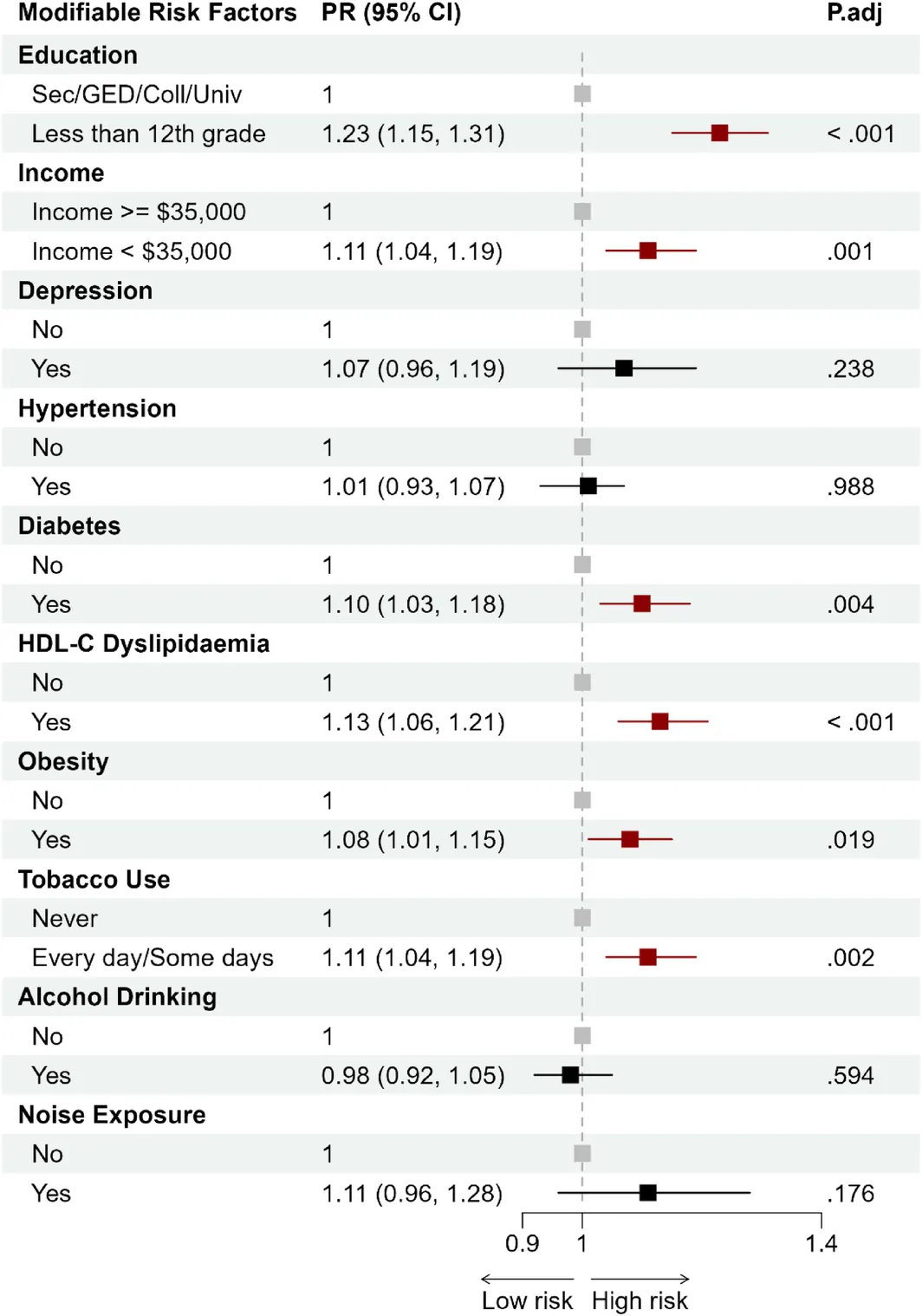
Associations of 10 modifiable risk factors with hearing loss. Hearing loss was defined as PTA ≥ 20 dB HL at four‐frequency PTA in the better ear. Four‐frequency PTA: mean hearing threshold across 0.5, 1, 2 and 4 kHz. The modified Poisson regression model was used to calculate the PRs. In addition to age, sex and ethnicity, the model was mutually adjusted for all other risk factors. CI: confidence interval; HDL‐C, high‐density lipoprotein cholesterol; PR: prevalence ratio; PTA, pure‐tone average.

### 3.3 Population Attributable Risks of 10 Modifiable Risk Factors With Hearing Loss

Over 25% of hearing loss cases in the population were attributed to modifiable risk factors, with socio‐economic risk factors being the most significant contributors (PAF: 13.6%), followed by metabolic risk factors (PAF: 8.7%) and behavioural risk factors (PAF: 3.2%). Among 10 modifiable risk factors, low educational attainment accounts for the largest (PAF: 7.9%), followed by low income (PAF: 5.8%), tobacco use (PAF: 3.2%), HDL‐C dyslipidaemia (PAF: 2.9%) and obesity (PAF: 2.8%) (Table 3).

**TABLE 3:** Contributions of modifiable risk factors to hearing loss using average population attributable fractions.^a^

Exposed category	Reference category	PAF (95% CI)^b^
Low education (less than 12th grade)	Intermediate or high education (high school/GED/college/university)	7.9 (6.0, 9.7)
Income < $35 000	Income ≥ $35 000	5.8 (2.5, 9.0)
Depression	No	0.5 (−0.5, 1.4)
Hypertension	No	0.1 (−3.7, 3.8)
Type 2 diabetes	No	3.0 (1.3, 4.7)
HDL‐C dyslipidaemia	No	2.9 (0.9, 4.9)
Obesity	No	2.8 (0.2, 5.4)
Tobacco use (every day/some days)	Never	3.2 (1.1, 5.3)
Alcohol drinking	No	−2.6 (−8.0, 2.8)
Noise exposure	No	0.5 (−0.3, 1.3)
Overall	—	25.5
Socio‐economic and psychological risks	/	13.6
Metabolic risks	/	8.7
Behavioural risks	/	3.2
Environmental risks	/	/

Abbreviations: CI: confidence interval; GED, general educational development; HDL‐C, high‐density lipoprotein cholesterol; PAF, population attributable fraction.

^a^ Hearing loss was defined as PTA ≥ 20 dB HL at four‐frequency PTA in the better ear. Four‐frequency PTA: mean hearing threshold across 0.5, 1, 2 and 4 kHz.

^b^ PAFs represent the proportion of hearing loss that would theoretically be prevented if the risk factor were eliminated; PAFs were mutually adjusted for 10 modifiable risk factors in addition to age, sex and ethnicity. The primary unweighted PAFs were derived from the *averisk* package.

### 3.4 Sensitivity Analysis

Similar associations were observed for hearing loss at high‐ and low‐frequency PTA (Table S1). When assessing the associations for moderate hearing loss (four‐frequency PTA > 40 dB HL), only low income was associated with moderate hearing loss (PR 1.37, 95% CI 1.05–1.78) (Table S2). Patterns of associations were different when stratified analyses were conducted, for example, when stratified by age (Table S3), diabetes was associated with hearing loss in middle‐aged adults (aged 40–65 years, PR 1.26, 95% CI 1.10–1.43), but not in older adults (aged ≥ 65 years); when stratified by sex (Table S4), diabetes was associated with hearing loss in women (PR 1.19, 95% CI 1.07–1.31), but not in men. Ethnic stratification revealed diverse associations across five ethnic categories (Table S5). For example, tobacco use was the only associated risk factor for hearing loss in Mexican Americans (PR 1.24, 95% CI 1.03–1.48). While in other Hispanics, low education (PR 1.52, 95% CI 1.21–1.89) and diabetes (PR 1.57, 95% CI 1.27–1.94) were associated with hearing loss. For non‐Hispanic Whites, hearing loss‐associated risk factors included low education (PR 1.34, 95% CI 1.22–1.47), low income (PR 1.15, 95% CI 1.05–1.25), HDL‐C dyslipidaemia (PR 1.12, 95% CI 1.02–1.23) and tobacco use (PR 1.14, 95% CI 1.04–1.25). Among non‐Hispanic Blacks, low education (PR 1.28, 95% CI 1.09–1.50) and low income (PR 1.18, 95% CI 1.01–1.39) were associated with hearing loss. In other races, low education (PR 1.50, 95% CI 1.16–1.95) and HDL‐C dyslipidaemia (PR 1.34, 95% CI 1.04–1.73) were suggested as associated risk factors of hearing loss.

The calculations of adjusted PAFs (Table S6) indicated similar estimates for the average PAFs, with the largest contribution to hearing loss of socio‐economic factors (PAF: 11.2%). Among these, low educational attainment was the largest risk factor, accounting for 6.2% of the PAF. When including all 10 414 individuals regardless of hearing threshold severity, association patterns (Table S7) remained largely consistent with the primary findings (Figure 2). Depression was the only factor that showed a differential pattern: it was significantly associated with severe to profound hearing loss (PR 1.09, 95% CI [1.01, 1.17]; Table S7) but not with mild to moderate hearing loss (PR 1.07, 95% CI [0.96, 1.19]; Figure 2). Weighted sensitivity analyses accounting for the complex survey design yielded broadly consistent results. Overall weighted PAF was 25.0% (calculated multiplicatively as 1 − product of (1 − *PAFᵢ*)), this is highly consistent with 25.5% of overall unweighted PAF, suggesting the robustness of PAF analysis. Top contributing factors remained, including low educational attainment (weighted PAF 4.7%), HDL‐C dyslipidaemia (weighted PAF 6.3%), obesity (weighted PAF 4.8%), tobacco use (weighted PAF 5.7%) and low income (weighted PAF 2.8%). The direction of associations (weighted PRs) remained consistent for all risk factors (Table S8).

## 4 Discussion

### 4.1 Principal Findings

Our results, aligning with previous studies, confirm that the prevalence of hearing loss at high‐frequency PTA is higher than at four‐ or low‐frequency PTA. We found that lower educational attainment, lower income, diabetes, HDL‐C dyslipidaemia, obesity and tobacco use were associated with hearing loss in adults aged 40–85 years. PAF analyses revealed that over 25% of hearing loss cases could be attributed to modifiable risk factors in both unweighted (25.5%) and survey‐weighted (25.0%) analyses. Low educational attainment, low income, HDL‐C dyslipidaemia, obesity and tobacco use emerged as main contributors, with some variation in ranking between unweighted and survey‐weighted analyses.

### 4.2 Socio‐Economic Risks

Our research suggested associations between low educational attainment, low income and hearing loss. Individuals with low socioeconomic status often adopt unhealthy lifestyles, such as low protection of hearing, excessive alcohol drinking and smoking, which can lead to worsening systemic atherosclerosis and hearing loss [18]. An alternative explanation may involve that less educated or low‐income individuals tend to live or work in noisy environments without any hearing protection [19].

It is noteworthy that while low educational attainment showed the highest PAF in unweighted analyses (7.9%), its contribution was attenuated in survey‐weighted analyses (4.7%). This discrepancy likely reflects the complex survey design and differential weighting of population subgroups. Nevertheless, socioeconomic factors remain important targets for public health interventions.

### 4.3 Metabolic Risks

Our study also identified diabetes as a risk factor for hearing loss, with age‐stratified analyses showing an impact on hearing in middle‐aged (aged 40–64 years) but not older adults (aged over 65 years). Hyperglycaemia may cause cochlear micro‐angiopathy and diabetic neuropathy, contributing to hearing loss through vascular and nerve damage [20].

HDL‐C dyslipidaemia was a major metabolic risk factor, with an unweighted PAF of 2.9% and a survey‐weighted PAF of 6.3%, emerging as the leading contributor in the weighted analysis. Potential mechanisms may involve dyslipidaemia‐induced vascular obstruction and endothelial dysfunction, which reduce blood flow and oxygen supply to the cochlea, ultimately impairing auditory function [11].

With regards to the observed positive associations between obesity and hearing loss, potential mechanisms may involve visceral adipose tissue (VAT). This acts as a metabolic, immunologic and endocrine organ, can induce systemic inflammation by secreting pro‐inflammatory molecules, contributing to chronic inflammation and accelerating ageing and such process increases the risk of age‐related diseases (e.g., hearing loss) [21, 22].

### 4.4 Behavioural and Environmental Risks

Tobacco use has been reported to be associated with peripheral vascular disturbances and elevation of blood pressure [23]. In our study, tobacco use was significantly associated with hearing loss (PR 1.11, 95% CI [1.04, 1.19]), though hypertension itself was not statistically significant after adjusting for other risk factors. Possible mechanisms for tobacco's effect include the direct ototoxic effect of nicotine or tobacco‐induced ischemia in the cochlea through the production of carboxyhaemoglobin, vasospasm and increased blood viscosity [24].

### 4.5 Strengths and Limitations

This study differs from previous research, which often focused on a limited set of risk factors for hearing loss. We evaluated multiple clusters of modifiable risk factors with mutual adjustments and assessed associations between multiple modifiable risk factors and hearing loss across different age, sex and ethnic categories. For association analyses, we used modified Poisson regression, an ideal model for cross‐sectional chronic disease studies, offering more robust PRs estimations than odds ratios [15, 25]. In the current investigation, PAFs are applied to measure the proportion of hearing loss that can be avoided by removing a modifiable risk factor at the population level. This is advantageous because PAFs can measure disease burden due to the highly correlated risk factors.

However, our study has limitations. Cross‐sectional design prevents causal inference, so follow‐up studies are needed to observe the development of hearing loss. Some risk factors may be underestimated (such as noise exposure and alcohol drinking), as they were assessed by the questionnaire. For example, ‘unhealthy’ lifestyle choices may be misrepresented due to social desirability, conformity bias or recall bias. Additionally, noise exposure was limited to self‐reported occupational exposure rather than recreational noise, preventing a comprehensive noise exposure assessment. Given the increasing prevalence of personal audio device use, future studies should incorporate detailed recreational noise exposure measurements. Moreover, the dichotomisation of income and education variables enhanced interpretability but may have attenuated multi‐category exposure and outcome associations. Last, risk factors like physical activity and diet were not included in this research as it is hard to evaluate based on the participant's recall. Future studies should incorporate these additional risk factors.

## 5 Conclusions

In conclusion, socio‐economic, metabolic and behavioural factors are associated with hearing loss. A substantial proportion (> 25%) of hearing loss cases could be prevented by addressing modifiable risk factors. Unweighted and survey‐weighted analyses highlighted low education and income, HDL‐C dyslipidaemia, tobacco use and obesity as main contributors. Therefore, hearing promotion policies and clinical interventions should adopt a multifaceted approach to reduce the health impacts of hearing loss.

## Author Contributions

**Yulu Zheng:** conceptualisation, data curation, formal analysis, investigation, methodology, resources, software, validation, visualisation, writing – original draft, writing – review and editing. **Tingting Li:** formal analysis, visualisation, writing – review and editing. **Zhiyuan Wu:** data curation, methodology, investigation. **Yahong Zhu:** data curation, validation, visualisation. **Zheng Guo:** conceptualisation, methodology, project administration, supervision, writing – review and editing. **Xingang Li:** conceptualisation, methodology, funding acquisition, project administration, supervision, writing – review and editing.

## Funding

This work was supported by the Western Australian Future Health Research and Innovation (FHRI) Fund through the WA Near‐Miss Awards Ideas Grants (WANMA IG) 2025–26 Program, the Australian Government Research Training Program (RTP) Scholarship and the ECU Stan Perron Foundation Top Up Scholarship funded by the Stan Perron Charitable Foundation Limited.

## Ethics Statement

The NHANES protocol was approved by the National Center for Health Statistics Ethics Review Board. Because this study utilised publicly available, de‐identified data files from the NHANES database, it was exempt from additional institutional review board approval.

## Consent

All study participants provided written informed consent prior to participating in the original NHANES data collection (https://www.cdc.gov/nchs/nhanes/irba98.htm). Informed consent was not required for this secondary data analysis due to the public and de‐identified nature of the dataset.

## Conflicts of Interest

The authors declare no conflicts of interest.

## Supporting information


**Data S1:** Supporting Information.
**Table S1:** Associations of 10 modifiable risk factors with hearing loss at high‐frequency PTA and low‐frequency PTA.
**Table S2:** Associations of 10 modifiable risk factors with moderate hearing loss.
**Table S3:** Associations of 10 modifiable risk factors with hearing loss, stratified by age.
**Table S4:** Associations of 10 modifiable risk factors with hearing loss, stratified by sex.
**Table S5:** Associations of 10 modifiable risk factors with hearing loss, stratified by ethnicity.
**Table S6:** Adjusted population attributable fractions for hearing loss.
**Table S7:** Associations of 10 modifiable risk factors with hearing loss among 10 414 adults.
**Table S8:** Weighted population attributable fractions and weighted prevalence ratios for hearing loss.

## Data Availability

Access to the raw data can be obtained from the National Health and Nutrition Examination Survey (https://www.cdc.gov/nchs/nhanes/index.htm). The cleaned and processed data used in this study can be requested from the corresponding author.
