# Supplementary material for: Modifiable Risk Factors and Hearing Loss: The National Health and Nutrition Examination Survey 1999–2020
Source: Clin Otolaryngol. 2026 Jul 31;51(6):929–37. doi: 10.1111/coa.70153 (PMC13631807; doi:10.1111/coa.70153)
Supplement: Supplementary file 1 [file COA-51-929-s001.docx]

**Supplementary Material**

**Modifiable Risk Factors and Hearing Loss: The National Health and Nutrition Examination Survey 1999-2020**

**Calculation of the prevalence ratio**

In this study, owing to the high prevalence of hearing loss among middle-aged and older people, prevalence ratio (PR) rather than odds ratio (OR) was used. Because OR tends to “overestimate” the strength of the cross-sectional association.

*The example* shows the explanations for OR and PR. Take a prevalent disease as an example, if 70 out of 100 exposed individuals have that disease, and 35 out of 100 non-exposed individuals suffer the same disease, then the OR is (70/30) / (35/65) = 4.3. However, the PR is (70/100) / (35/100) = 2.0. This suggests that PR should be used in the cross-sectional design, and “OR” could only be used as an estimation of PR when the prevalence of the disease is “rare” (e.g., <5%) [1-3].

**Example:**

|  | ***Outcome*** | |  |
| --- | --- | --- | --- |
| ***Exposure*** | Yes | No | Total |
| Yes | 70 (a) | 30 (b) | 100 (a+b) |
| No | 35 (c) | 65 (d) | 100 (c+d) |
| Total | 105 (a+c) | 95 (b+d) | 200 (N) |

Prevalence ratio (PR): Odds ratio (OR):

Outcome = “Yes”: $\frac{a}{a+b}$/ $\frac{c}{c+d}$ Outcome = “Yes”: $\frac{a}{c}$ / $\frac{b}{d}$= (ad / bc)

**Calculation of the average population attributable fractions as excerpted from previous studies [4-6]**

Average population attributable fractions (APAFs) and their 95% confidence intervals were calculated using the approach described by Eide and Olaf. In this approach, APAF for each risk factor is based on logistic regression, modifiable risk factors of interest are added to the model in every possible order, and the average of all PAFs is then calculated using the R package ‘*averisk’* developed by Ferguson et. al. Estimates of PAF were further adjusted for age and sex.

The adjusted attributable fraction for each disease can be estimated by calculating the predicted probabilities of a modifiable risk factor for each participant, assuming that the participant is unexposed to the specific risk factor. Under this assumption, a probabilistic model for hearing loss with adjustment of risk factors and potential confounding factors is needed. Based on the logistic model, calculation of the predicted probability of risk factors for participant $i{( \hat{p}}_{i})$ is provided in *Equation (1)*.

$\hat{p}_{i} =\frac{1}{1 + exp(-(\hat{\alpha}+ \sum_{j} \hat{\beta}xij))}$	(1)

Where $\hat{\alpha}i$ is the estimated intercept of the logistic regression model, $xij$ represents a dummy variable (zero or one) for the existence of risk factor $j$ **within participant** $i$ **and** $\hat{\beta}$ **the parameter vector for the modifiable risk factor included in the model. An estimator of adjusted PAF for the burden of hearing loss due to modifiable risk factor** $j$ **can be derived by calculating predicted probabilities from** *Equation (1)***, setting** $xij$ **to zero for all participants, and leaving the values of the other covariates unchanged. Then, adjusted PAF is then calculated by** *Equation (2)*.

$Adj.PAF =\frac{O-E}{O}$	(2)

$E$ **is the sum of these predicted probabilities:** $E=Ewipi$ **,** $wi$ **the survey weight, is proportional to the expected number of participants with hearing loss that may be observed if the risk factor was removed from the population. The total weighted observed cases** $O=Ewiyi$**, with** $yi$ **being the indicator function for hearing loss in participant** $i$**. APAFs present with adjusted PAFs, by averaging all sequential AFs for the same risk factor, corresponding to all elimination orders.**

**Table S1. Associations of 10 modifiable risk factors with hearing loss at high-frequency PTA and low-frequency PTA ^a^**

| **Variable** | **Hearing Loss, prevalence ratio (95% CI) ^b^** | | **Hearing Loss, prevalence ratio (95% CI) ^b^** | |
| --- | --- | --- | --- | --- |
|  | **High-frequency PTA** | **P-value Adjustment** | **Low-frequency PTA** | **P-value Adjustment** |
| **Education** |  |  |  |  |
| Sec/GED/Coll/Uni | 1 |  | 1 |  |
| Less than 12th grade | 1.12 (1.08, 1.16) | < .001 | 1.32 (1.21, 1.44) | < .001 |
| **Income** |  |  |  |  |
| Income >= $35,000 | 1 |  | 1 |  |
| Income < $35,000 | 1.06 (1.02, 1.09) | < .001 | 1.09 (1.01, 1.18) | .046 |
| **Depression** |  |  |  |  |
| No | 1 |  | 1 |  |
| Yes | 1.06 (1.01, 1.13) | .034 | 1.11 (0.96, 1.27) | .147 |
| **Hypertension** |  |  |  |  |
| No | 1 |  | 1 |  |
| Yes | 1.02 (0.98, 1.05) | .362 | 1.01 (0.92, 1.10) | .910 |
| **Diabetes** |  |  |  |  |
| No | 1 |  | 1 |  |
| Yes | 1.06 (1.03, 1.10) | < .001 | 1.16 (1.06, 1.27) | < .001 |
| **HDL-C Dyslipidaemia** |  |  |  |  |
| No | 1 |  | 1 |  |
| Yes | 1.04 (1.01, 1.08) | .018 | 1.09 (0.99, 1.19) | .054 |
| **Obesity** |  |  |  |  |
| No | 1 |  | 1 |  |
| Yes | 1.05 (1.02, 1.09) | .003 | 1.09 (1.01, 1.18) | .048 |
| **Tobacco Use** |  |  |  |  |
| Never | 1 |  | 1 |  |
| Every day/Some days | 1.08 (1.04, 1.12) | < .001 | 1.10 (1.01, 1.20) | .044 |
| **Alcohol Drinking** |  |  |  |  |
| No | 1 |  | 1 |  |
| Yes | 1.00 (0.97, 1.04) | .899 | 0.97 (0.89, 1.06) | .504 |
| **Noise Exposure** |  |  |  |  |
| No | 1 |  | 1 |  |
| Yes | 1.01 (0.93, 1.09) | .840 | 1.12 (0.91, 1.37) | .280 |

*Abbreviations*: PR: prevalence ratio; CI: confidence interval; GED, General Educational Development; HDL-C, high density lipoprotein cholesterol; PTA, pure-tone average.

^a^ Hearing loss was defined as high-frequency PTA or low-frequency PTA ≥ 20 dB HL (better ear). High-frequency PTA, mean hearing threshold across 3, 4, 6, and 8 kHz. Low-frequency PTA, mean hearing threshold across 0.5, 1, and 2 kHz frequency.

^b^ Modified Poisson regression model was used to calculate the prevalence ratios. In addition to age, sex, and ethnicity, the model was mutually adjusted for all other risk factors.

**Table S2. Associations of 10 modifiable risk factors with moderate hearing loss ^a^**

| **Variable** | **Hearing Loss, prevalence ratio (95% CI) ^b^** | **P-value Adjustment** |
| --- | --- | --- |
| **Education** |  |  |
| Sec/GED/Coll/Uni | 1 |  |
| Less than 12th grade | 1.22 (0.94, 1.59) | .14 |
| **Income** |  |  |
| Income >= $35,000 | 1 |  |
| Income < $35,000 | 1.37 (1.05, 1.78) | .02 |
| **Depression** |  |  |
| No | 1 |  |
| Yes | 1.40 (0.96,2.04) | .08 |
| **Hypertension** |  |  |
| No | 1 |  |
| Yes | 0.88 (0.67,1.16) | .38 |
| **Diabetes** |  |  |
| No | 1 |  |
| Yes | 1.11 (0.84,1.47) | .46 |
| **HDL-C Dyslipidaemia** |  |  |
| No | 1 |  |
| Yes | 1.13 (0.86,1.49) | .39 |
| **Obesity** |  |  |
| No | 1 |  |
| Yes | 0.89 (0.68,1.16) | .37 |
| **Tobacco Use** |  |  |
| Never | 1 |  |
| Every day/Some days | 1.09 (0.83,1.43) | .55 |
| **Alcohol Drinking** |  |  |
| No | 1 |  |
| Yes | 0.99(0.77,1.27) | .92 |
| **Noise Exposure** |  |  |
| No | 1 |  |
| Yes | 1.71(0.99,2.96) | .05 |

*Abbreviations*: PR: prevalence ratio; CI: confidence interval; GED, General Educational Development; HDL-C, high density lipoprotein cholesterol; PTA, pure-tone average.

^a^ Hearing loss was defined as four-frequency PTA or low-frequency PTA > 40 dB HL (better ear). Four-frequency PTA: mean hearing threshold across 0.5, 1, 2, and 4 kHz.

^b^ Modified Poisson regression model was used to calculate the prevalence ratios. In addition to age, sex, and ethnicity, the model was mutually adjusted for all other risk factors.

**Table S3. Associations of 10 modifiable risk factors with hearing loss, stratified by age ^a^**

| **Variable** | **Hearing Loss, prevalence ratio (95% CI) ^b^** | | **Hearing Loss, prevalence ratio (95% CI) ^b^** | |
| --- | --- | --- | --- | --- |
|  | **Aged 40-64 years** | **P-value Adjustment** | **Aged 65-85 years** | **P-value Adjustment** |
| **Education** |  |  |  |  |
| Sec/GED/Coll/Uni | 1 |  | 1 |  |
| Less than 12th grade | 1.32 (1.16,1.49) | < .001 | 1.17 (1.09,1.25) | < .001 |
| **Income** |  |  |  |  |
| Income >= $35,000 | 1 |  | 1 |  |
| Income < $35,000 | 1.15 (1.02, 1.30) | .02 | 1.07 (0.99, 1.15) | .06 |
| **Depression** |  |  |  |  |
| No | 1 |  | 1 |  |
| Yes | 1.15 (0.97,1.37) | .11 | 0.99 (0.87,1.12) | .87 |
| **Hypertension** |  |  |  |  |
| No | 1 |  | 1 |  |
| Yes | 1.02 (0.90,1.15) | .76 | 0.94 (0.87, 1.02) | .13 |
| **Diabetes** |  |  |  |  |
| No | 1 |  | 1 |  |
| Yes | 1.26 (1.10,1.43) | < .001 | 1.03 (0.95,1.10) | .49 |
| **HDL-C Dyslipidaemia** |  |  |  |  |
| No | 1 |  | 1 |  |
| Yes | 1.16 (1.03,1.31) | .01 | 1.12 (1.04,1.20) | < .001 |
| **Obesity** |  |  |  |  |
| No | 1 |  | 1 |  |
| Yes | 1.09 (0.97,1.22) | .14 | 1.06 (0.98,1.14) | .12 |
| **Tobacco Use** |  |  |  |  |
| Never | 1 |  | 1 |  |
| Every day/Some days | 1.17 (1.05,1.32) | .01 | 1.04 (0.97,1.13) | .28 |
| **Alcohol Drinking** |  |  |  |  |
| No | 1 |  | 1 |  |
| Yes | 0.94 (0.80,1.10) | .41 | 1.04 (0.97,1.13) | .66 |
| **Noise Exposure** |  |  |  |  |
| No | 1 |  | 1 |  |
| Yes | 1.13 (0.92, 1.38) | .23 | 1.04 (0.97,1.13) | .39 |

*Abbreviations*: PR: prevalence ratio; CI: confidence interval; GED, General Educational Development; HDL-C, high density lipoprotein cholesterol; PTA, pure-tone average.

^a^ Hearing loss was defined as four-frequency PTA or low-frequency PTA ≥ 20 dB HL (better ear). Four-frequency PTA: mean hearing threshold across 0.5, 1, 2, and 4 kHz.

^b^ Modified Poisson regression model was used to calculate the prevalence ratios. In addition to sex and ethnicity, the model was mutually adjusted for all other risk factors.

**Table S4. Associations of 10 modifiable risk factors with hearing loss, stratified by sex ^a^**

| **Variable** | **Hearing Loss, prevalence ratio (95% CI) ^b^** | | **Hearing Loss, prevalence ratio (95% CI) ^b^** | |
| --- | --- | --- | --- | --- |
|  | **Male** | **P-value Adjustment** | **Female** | **P-value Adjustment** |
| **Education** |  |  |  |  |
| Sec/GED/Coll/Uni | 1 |  | 1 |  |
| Less than 12th grade | 1.30 (1.19, 1.42) | <.001 | 1.14 (1.04, 1.26) | .01 |
| **Income** |  |  |  |  |
| Income >= $35,000 | 1 |  | 1 |  |
| Income < $35,000 | 1.11 (1.02, 1.21) | .02 | 1.11 (1.01, 1.22) | .04 |
| **Depression** |  |  |  |  |
| No | 1 |  | 1 |  |
| Yes | 1.03 (0.89, 1.18) | .71 | 1.13 (0.96, 1.32) | .15 |
| **Hypertension** |  |  |  |  |
| No | 1 |  | 1 |  |
| Yes | 1.05 (0.96, 1.15) | .31 | 0.92 (0.83, 1.03) | .15 |
| **Diabetes** |  |  |  |  |
| No | 1 |  | 1 |  |
| Yes | 1.05 (0.96, 1.15) | .25 | 1.19 (1.07, 1.31) | <.001 |
| **HDL-C Dyslipidaemia** |  |  |  |  |
| No | 1 |  | 1 |  |
| Yes | 1.13 (1.03, 1.25) | .01 | 1.14 (1.04, 1.26) | .01 |
| **Obesity** |  |  |  |  |
| No | 1 |  | 1 |  |
| Yes | 1.14 (1.04, 1.24) | <.001 | 1.03 (0.94, 1.13) | .52 |
| **Tobacco Use** |  |  |  |  |
| Never | 1 |  | 1 |  |
| Every day/Some days | 1.10 (0.99, 1.21) | .05 | 1.11 (1.01, 1.22) | .03 |
| **Alcohol Drinking** |  |  |  |  |
| No | 1 |  | 1 |  |
| Yes | 1.03 (0.94, 1.14) | .52 | 0.93 (0.85, 1.02) | .11 |
| **Noise Exposure** |  |  |  |  |
| No | 1 |  | 1 |  |
| Yes | 1.18 (0.99, 1.40) | .07 | 0.93 (0.71, 1.22) | .59 |

*Abbreviations*: PR: prevalence ratio; CI: confidence interval; GED, General Educational Development; HDL-C, high density lipoprotein cholesterol; PTA, pure-tone average.

^a^ Hearing loss was defined as four-frequency PTA or low-frequency PTA ≥ 20 dB HL (better ear). Four-frequency PTA: mean hearing threshold across 0.5, 1, 2, and 4 kHz.

^b^ Modified Poisson regression model was used to calculate the prevalence ratios. In addition to age and ethnicity, the model was mutually adjusted for all other risk factors.

**Table S5. Associations of 10 modifiable risk factors with hearing loss, stratified by ethnicity ^a^**

| **Exposed Category** | **Reference Category** | **Hearing loss, prevalence ratio (95% CI) ^b^** | | | | |
| --- | --- | --- | --- | --- | --- | --- |
|  |  | **Mexican American** | **Other Hispanic** | **Non-Hispanic White** | **Non-Hispanic Black** | **Other Race** |
| Low education (Less than 12th grade) | Intermediate or high education (Sec/GED/Coll/Uni) | 1.06 (0.89, 1.27) | **1.52 (1.21, 1.89)** | **1.34 (1.22, 1.47)** | **1.28 (1.09, 1.50)** | **1.50 (1.16, 1.95)** |
| Low income (Income < $35,000) | Middle or high income (Income >= $35,000) | 1.10 (0.92, 1.32) | 1.14 (0.90, 1.44) | **1.15 (1.05, 1.25)** | **1.19 (1.01, 1.39)** | 1.01 (0.78, 1.32) |
| Depression | No | 1.27 (0.99, 1.61) | 0.84 (0.57, 1.23) | 1.07 (0.93, 1.24) | 0.94 (0.71, 1.24) | 1.03 (0.67, 1.59) |
| Hypertension | No | 0.99 (0.83, 1.19) | 1.04 (0.81, 1.34) | 1.04 (0.95, 1.14) | 0.98 (0.79, 1.22) | 1.07 (0.81, 1.41) |
| Diabetes | No | 1.15 (0.97, 1.37) | **1.57 (1.27, 1.94)** | 1.09 (0.99, 1.19) | 1.03 (0.88, 1.21) | 1.18 (0.90, 1.53) |
| HDL-C Dyslipidaemia | No | 1.09 (0.91, 1.30) | 0.93 (0.74, 1.16) | **1.12 (1.02, 1.23)** | 1.13 (0.95, 1.35) | **1.34 (1.04, 1.73)** |
| Obesity | No | 1.11 (0.94, 1.31) | 1.04 (0.83, 1.31) | 1.09 (0.99, 1.19) | 1.12 (0.96, 1.31) | 1.15 (0.86, 1.54) |
| Tobacco Use (Every day/ Some days) | Never | **1.24 (1.03, 1.48)** | 1.13 (0.90, 1.41) | **1.14 (1.04, 1.25)** | 1.05 (0.90, 1.23) | 0.94 (0.71, 1.23) |
| Alcohol Drinking | No | 0.87 (0.71, 1.07) | 1.03 (0.81, 1.31) | 0.97 (0.89, 1.06) | 0.97 (0.83, 1.14) | 0.99 (0.76, 1.28) |
| Noise Exposure | No | 1.12 (0.77, 1.63) | 0.72 (0.39, 1.32) | 1.13 (0.93, 1.38) | 1.10 (0.74, 1.65) | 1.47 (0.96, 2.25) |

*Abbreviations*: PR: prevalence ratio; CI: confidence interval; GED, General Educational Development; HDL-C, high density lipoprotein cholesterol; PTA, pure-tone average.

^a^ Hearing loss was defined as four-frequency PTA or low-frequency PTA ≥ 20 dB HL (better ear). Four-frequency PTA: mean hearing threshold across 0.5, 1, 2, and 4 kHz.

^b^ Modified Poisson regression model was used to calculate the prevalence ratios. In addition to age and sex, the model was mutually adjusted for all other risk factors.

**Table S6. Adjusted population attributable fractions for hearing loss ^a^**

| **Exposed Category** | **Reference Category** | **Hearing Loss PAF (95% CI) ^b^** |
| --- | --- | --- |
| Low education (less than 12th grade) | Intermediate or high education (High school/College/University) | 6.2 (4.2, 8.3) |
| Income < $35,000 | Income ≥ $35,000 | 4.9 (1.8, 8.1) |
| Depression | No | 0.6 (-0.4, 1.6) |
| Hypertension | No | -1.9 (-6.1, 2.3) |
| Type 2 diabetes | No | 2.3 (0.5, 4.0) |
| HDL-C Dyslipidaemia | No | 3.9 (1.8, 5.9) |
| Obesity | No | 2.5 (-0.1, 5.1) |
| Tobacco Use (Every day/Some days) | Never | 3.1 (0.9, 5.3) |
| Alcohol Drinking | No | -2.1 (-7.7, 3.5) |
| Noise exposure | No | 0.6 (-0.1, 1.3) |
| Overall | - | 20.4 |
| Socio-economic and Psychological risks | / | 11.2 |
| Metabolic risks | / | 6.1 |
| Behavioural risks | / | 3.1 |
| Environmental risks | / | / |

*Abbreviations*: PAF, population attributable fractions; CI, confidence interval; GED, General Educational Development; HDL-C, high density lipoprotein cholesterol.

^a^ Hearing loss was defined as Four-frequency PTA ≥ 20 dB HL in the better ear.

^b^ Population attributable fractions were mutually adjusted for 10 modiﬁable risk factors in addition to age, sex, and ethnicity.

**Table S7. Associations of 10 modifiable risk factors with hearing loss among 10,414 adults ^a^**

| **Variable** | **Hearing Loss, prevalence ratio (95% CI) ^b^** | **P-value Adjustment** |
| --- | --- | --- |
| **Education** |  |  |
| Sec/GED/Coll/Uni | 1 |  |
| Less than 12th grade | 1.15 (1.10, 1.20) | <.001 |
| **Income** |  |  |
| Income >= $35,000 | 1 |  |
| Income < $35,000 | 1.10 (1.05, 1.14) | <.001 |
| **Depression** |  |  |
| No | 1 |  |
| Yes | 1.09 (1.01, 1.17) | .02 |
| **Hypertension** |  |  |
| No | 1 |  |
| Yes | 1.02 (0.97, 1.07) | .48 |
| **Diabetes** |  |  |
| No | 1 |  |
| Yes | 1.07 (1.02, 1.12) | .003 |
| **HDL-C Dyslipidaemia** |  |  |
| No | 1 |  |
| Yes | 1.11 (1.06, 1.17) | <.001 |
| **Obesity** |  |  |
| No | 1 |  |
| Yes | 1.07 (1.02, 1.11) | .004 |
| **Tobacco Use** |  |  |
| Never | 1 |  |
| Every day/Some days | 1.09 (1.04, 1.15) | <.001 |
| **Alcohol Drinking** |  |  |
| No | 1 |  |
| Yes | 0.99 (0.95, 1.03) | .59 |
| **Noise Exposure** |  |  |
| No | 1 |  |
| Yes | 1.10 (0.99, 1.22) | .06 |

*Abbreviations*: PR: prevalence ratio; CI: confidence interval; GED, General Educational Development; HDL-C, high density lipoprotein cholesterol; PTA, pure-tone average.

^a^ Hearing loss was defined as PTA ≥ 20 dB HL at four-frequency PTA in the better ear, including ≥ 70 dB HL. Four-frequency PTA: mean hearing threshold across 0.5, 1, 2, and 4 kHz.

^b^ Modified Poisson regression model was used to calculate the prevalence ratios. In addition to age, sex, and ethnicity, the model was mutually adjusted for all other risk factors.

**Table S8. Weighted population attributable fractions and weighted prevalence ratios for hearing loss ^a^**

| **Exposed Category** | **Reference Category** | **Weighted PAF (95% CI) ^b^** | **Weighted PR (95% CI) ^c^** |
| --- | --- | --- | --- |
| Low education (less than 12th grade) | Intermediate or high education (High school/ GED/College/University) | 4.7 (2.8, 6.6) | **1.31 (1.19, 1.45)** |
| Income < $35,000 | Income ≥ $35,000 | 2.8 (0.2, 5.5) | **1.09 (1.01, 1.18)** |
| Depression | No | 0.6 (-0.6, 2.0) | 1.09 (0.93, 1.28) |
| Hypertension | No | 2.8 (-2.3, 7.8) | 1.06 (0.95, 1.18) |
| Type 2 diabetes | No | 1.5 (-0.3, 3.4) | 1.10 (0.98, 1.24) |
| HDL-C Dyslipidaemia | No | 6.3 (3.1, 9.5) | **1.23 (1.11, 1.36)** |
| Obesity | No | 4.8 (1.4, 8.2) | **1.13 (1.04, 1.23)** |
| Tobacco Use (Every day/Some days) | Never | 5.7 (2.6, 8.8) | **1.18 (1.08, 1.29)** |
| Alcohol Drinking | No | -1.7 (-11.2, 7.1) | 0.98 (0.88, 1.09) |
| Noise exposure | No | 0.5 (-1.3, 2.8) | 1.07 (0.83, 1.38) |

*Abbreviations*: PR: prevalence ratio; PAF: population attributable fractions; CI: confidence interval; GED, General Educational Development; HDL-C, high density lipoprotein cholesterol; PTA, pure-tone average.

^a^ Hearing loss was defined as PTA ≥ 20 dB HL at four-frequency PTA in the better ear, including ≥ 70 dB HL. Four-frequency PTA: mean hearing threshold across 0.5, 1, 2, and 4 kHz.

^b^ Weighted PAFs were approximated using Levin’s formula based on the weighted prevalence ratios from survey-weighted modified Poisson regression models.

^c^ Weighted PRs were estimated from survey-weighted modified Poisson regression models (quasipoisson family with log link), mutually adjusted for all other modifiable risk factors, age, gender, and ethnicity, taking into account the complex sampling design of NHANES (including MEC weights, strata, and primary sampling units).

Statistically significant associations were indicated in **bold**.

**References**

1.	Lee J, Chuen ST, Kee SC. A practical guide for multivariate analysis of dichotomous outcomes. Ann Acad Med Singapore 2009;38(8):714-719. https://doi.org/10.47102/annals-acadmedsg.v38n8p714.

2.	Martinez BAF, Leotti VB, Silva GdSe, Nunes LN, Machado G, Corbellini LG. Odds Ratio or Prevalence Ratio? An Overview of Reported Statistical Methods and Appropriateness of Interpretations in Cross-sectional Studies with Dichotomous Outcomes in Veterinary Medicine. Frontiers in Veterinary Science 2017;4https://doi.org/10.3389/fvets.2017.00193.

3.	Tamhane AR, Westfall AO, Burkholder GA, Cutter GR. Prevalence odds ratio versus prevalence ratio: choice comes with consequences. Statistics in Medicine 2016;35(30):5730-5735. https://doi.org/10.1002/sim.7059.

4.	Yusuf S, Joseph P, Rangarajan S, et al. Modifiable risk factors, cardiovascular disease, and mortality in 155 722 individuals from 21 high-income, middle-income, and low-income countries (PURE): a prospective cohort study. The Lancet 2020;395(10226):795-808. https://doi.org/10.1016/S0140-6736(19)32008-2.

5.	Li S, Liu Z, Joseph P, et al. Modifiable risk factors associated with cardiovascular disease and mortality in China: a PURE substudy. Eur Heart J 2022;43(30):2852-2863. https://doi.org/10.1093/eurheartj/ehac268.

6.	Palazzo C, Yokota RT, Ferguson J, et al. Methods to assess the contribution of diseases to disability using cross-sectional studies: comparison of different versions of the attributable fraction and the attribution method. Int J Epidemiol 2019;48(2):559-570. https://doi.org/10.1093/ije/dyy222.
